# Occurrence, Phenotypic and Molecular Characteristics of Extended-Spectrum Beta-Lactamase-Producing *Escherichia coli* in Healthy Turkeys in Northern Egypt

**DOI:** 10.3390/antibiotics11081075

**Published:** 2022-08-08

**Authors:** Amira A. Moawad, Helmut Hotzel, Hafez M. Hafez, Hazem Ramadan, Herbert Tomaso, Sascha D. Braun, Ralf Ehricht, Celia Diezel, Dominik Gary, Ines Engelmann, Islam M. Zakaria, Reem M. Reda, Samah Eid, Momtaz A. Shahien, Heinrich Neubauer, Stefan Monecke

**Affiliations:** 1 Friedrich-Loeffler-Institut, Institute of Bacterial Infections and Zoonoses, Naumburger Str. 96a, 07743 Jena, Germany; 2 Animal Health Research Institute, Agriculture Research Center (ARC), Giza 12618, Egypt; 3 Institute for Poultry Diseases, Free University Berlin, Königsweg 63, 14163 Berlin, Germany; 4 Hygiene and Zoonoses Department, Faculty of Veterinary Medicine, Mansoura University, Mansoura 35516, Egypt; 5 Leibniz Institute of Photonic Technology (IPHT), 07745 Jena, Germany; 6 InfectoGnostics Research Campus Jena e.V., Philosophenweg 7, 07743 Jena, Germany; 7 Institute of Physical Chemistry, Friedrich -Schiller University, 07743 Jena, Germany; 8 INTER-ARRAY by fzmb GmbH, 99947 Bad Langensalza, Germany; 9 BLINK AG, 07747 Jena, Germany; 10 Institute for Medical Microbiology and Virology, Dresden University Hospital, Fetscher Str. 74, 01307 Dresden, Germany

**Keywords:** colistin, ESBL, carbapenemases, *E. coli*, Turkey (*Meleagris gallopavo*), microarray, Egypt

## Abstract

Poultry is one of the most important reservoirs for zoonotic multidrug-resistant pathogens. The indiscriminate use of antimicrobials in poultry production is a leading factor for development and dissemination of antimicrobial resistance. This study aimed to describe the prevalence and antimicrobial resistance of *E. coli* isolated from healthy turkey flocks of different ages in Nile delta region, Egypt. In the current investigation, 250 cloacal swabs were collected from 12 turkey farms in five governorates in the northern Egypt. Collected samples were cultivated on Brilliance^TM^ ESBL agar media supplemented with cefotaxime (100 mg/L). The *E. coli* isolates were identified using MALDI-TOF-MS and confirmed by a conventional PCR assay targeting 16S rRNA-DNA. The phenotypic antibiogram against 14 antimicrobial agents was determined using the broth micro-dilution method. DNA-microarray-based assay was applied for genotyping and determination of both, virulence and resistance-associated gene markers. Multiplex real-time PCR was additionally applied for all isolates for detection of the actual most relevant Carbapenemase genes. The phenotypic identification of colistin resistance was carried out using E-test. A total of 26 *E. coli* isolates were recovered from the cloacal samples. All isolates were defined as multidrug-resistant. Interestingly, two different *E. coli* strains were isolated from one sample. Both strains had different phenotypic and genotypic profiles. All isolates were phenotypically susceptible to imipenem, while resistant to penicillin, rifampicin, streptomycin, and erythromycin. None of the examined carbapenem resistance genes was detected among isolates. At least one beta-lactamase gene was identified in most of isolates, where *bla*TEM was the most commonly identified determinant (80.8%), in addition to *bla*CTX-M9 (23.1%), *bla*SHV (19.2%) and *bla*OXA-10 (15.4%). Genes associated with chloramphenicol resistance were *flo*R (65.4%) and *cml*A1 (46.2%). Tetracycline- and quinolone-resistance-associated genes *tet*A and *qnr*S were detected in (57.7%) and (50.0%) of isolates, respectively. The aminoglycoside resistance associated genes *aad*A1 (65.4%), *aad*A2 (53.8%), *aph*A (50.0%), *str*A (69.2%), and *str*B (65.4%), were detected among isolates. Macrolide resistance associated genes *mph* and *mrx* were also detected in (53.8%) and (34.6%). Moreover, colistin resistance associated gene *mcr*-9 was identified in one isolate (3.8%). The class 1 integron integrase *int*I1 (84.6%), transposase for the transposon *tnp*ISEcp1 (34.6%) and OqxB -integral membrane and component of RND-type multidrug efflux pump *oqx*B (7.7%) were identified among the isolates. The existing high incidence of ESBL/colistin-producing *E. coli* identified in healthy turkeys is a major concern that demands prompt control; otherwise, such strains and their resistance determinants could be transmitted to other bacteria and, eventually, to people via the food chain.

## 1. Introduction

The increasing incidence of intestinal colonization with extended-spectrum β-lactamase (ESBL)-producing *Enterobacteriaceae* observed in food animals including poultry, emphasizes the possibility that food animals are major sources of ESBL-producing bacteria. This can result in an emerging public health hazard due to the compromised efficacy of the treatment of infectious diseases in humans and animals [1,2,3,4]. ESBL producing bacteria have been recovered from livestock (swine, cattle, poultry and turkey), from companion animals (cats, dogs and horses) and from wild animals [5]. The continuous exposure of bacteria to β-lactams as the most extensively used group of antibiotics in the world at the mean time has led to massive proliferation and extensive evolution of β-lactamases [6]. The ESBLs are those that hydrolyze third generation cephalosporins. Some are derived from mutations in the *bla*TEM-1 and *bla*SHV-1 *β*-lactamase genes, while others originate from horizontal gene transfer from environmental bacteria, such as *bla*CTX-M enzymes from *Kluyvera* species. ESBLs are mainly linked to the family of *Enterobacteriaceae*, including *Klebsiella* spp., *Salmonella enterica*, *Citrobacter* spp., *Enterobacter* spp., *Serratia* spp. and *Escherichia coli* [6]. Many ESBL-producers are also resistant to non-β-lactam antibiotics, including aminoglycosides, fluoroquinolones, tetracyclines, trimethoprim, sulfonamides, and chloramphenicol [7,8]. 

This concern encouraged the European Union (EU) to forbid the use of antimicrobials as growth promoters in food animals [9]. However, in some countries including Egypt, antimicrobials are still used in the poultry industry for non-therapeutic purposes such as prevention of diseases and promotion of growth [10,11,12]. ESBLs has been described in healthy poultry, in broiler chicken fecal and cecal samples and in turkey flocks [13,14,15]. In Egypt, *Escherichia* (*E.*) *coli* infections are considered one of the most serious infections causing economic losses in poultry production [16]. Despite the huge economic size of the Egyptian poultry industry, turkey production is still considered a growing sector limited to a small scale. Many recent studies have discussed the presence of ESBL producing *E. coli* in chickens in Egypt, while very few data are available about the same problem in turkeys [17,18]. 

Carbapenems are considered the last-line agents against ESBL-producing *Enterobacteriaceae.* The carbapenem resistance is either due to carbapenemase production, decrease in bacterial outer membrane permeability with production of AmpC/ESBL, or due to the efflux pumps [19]. Carbapenemases are beta-lactamases having high affinity for hydrolysis of penicillins, cephalosporins, and carbapenems [20]. Carbapenem-resistant *Enterobacteriaceae* (CRE) including *E. coli*, have gained a great attention since their outbreaks in the last two decades [21]. Infections caused by CRE cause high fatality rates in humans due to their resistance against the whole class of beta lactam antibiotics [22,23]. Carbapenemases are usually associated with resistances against other antimicrobial classes such as β-lactamases, aminoglycosides, and fluoroquinolones leaving very limited treatment options. Additionally, carbapenemase-encoding genes are easily transferable through mobile genetic elements as, e.g., plasmids and transposons, and spread easily among *Enterobacteriaceae* [24]. The CRE were classified as “critical priority pathogens” by World Health Organization [WHO] in 2017 [25]

Common carbapenemase genes include oxacillinase-48-like [OXA-48], *Klebsiella pneumoniae* carbapenemase [KPC], and the New Delhi metallo-β-lactamase [NDM].

Less common genes include imipenem-resistant Pseudomonas (IMP)-type carbapenemases, VIM (Verona integron-encoded metallo-β-lactamase), SIM (Seoul imipenemase), and GIM (German imipenemase) [19].

Colistin is considered the drug of choice in veterinary medicine for the treatment of recurrent *E. coli*-related digestive tract infections in animals used for food production. The drug is among the last-resort antimicrobials for the treatment of diseases caused by MDR Gram-negative bacteria. The rising rate of colistin resistance is mainly attributed to the use of the drug in veterinary medicine. The evolution of colistin resistance has recently raised serious concerns and the plasmid-borne *mcr* gene has been implicated in resistance in *Enterobacteriaceae* around the globe [17,26].

Many different single PCRs or phenotypic tests are available to obtain information about the different genetic features of *E. coli* isolates. More extensive information about the bacterial genotype can be obtained by DNA microarrays, which allow the parallel identification of a variety of genes. The microarray includes many oligonucleotide probes in total, covering various genes for clinically relevant features and typing.

The objectives of this study were to estimate the prevalence and antimicrobial resistance (AMR) in *E. coli* isolates originating from healthy turkey flocks in different districts in northern Egypt and to understand its public health significance. In addition, ESBL, Carbapenemase, and colistin producing *E. coli* and the possibility of their transmission to humans were investigated.

## 2. Materials and Methods

### 2.1. Isolation and Characterisation of E. coli Strains

A total of 250 cloacal swabs were randomly obtained from healthy turkeys housed in 12 farms spread over five governorates in the Nile Delta of Egypt, namely Dakahlia, Damietta, Kafr El-Sheikh, Sharkiya, and Gharbiya. Table 1 provides information about the studied poultry farms, the number of birds and the number of samples that were collected. Sterile swabs were used to carry out the sampling. The samples were subjected to microbiological analysis in the laboratory directly after being transported at a temperature of 4 °C. The samples were enriched using buffered peptone water. In order to identify ESBL-producing *E. coli*, after 24 h incubation at 37 °C cultures were streaked on Brilliance™ ESBL agar (Oxoid GmbH, Wesel, Germany), a commercial selective media that contains cefpodoxime (100 mg/L).

### 2.2. Identification by MALDI-TOF MS

Recovered isolates were identified using the MALDI-TOF MS Ultraflex instrument (BrukerDaltonics GmbH, Bremen, Germany) as described previously [27,28].

### 2.3. Phenotypic Testing for Antimicrobial Susceptibility

All isolates were tested for antimicrobial susceptibility with the MICRONAUT system (Merlin, Bornheim, Germany) using commercial 96-well microtiter plates as per manufacturer’s instructions. With the use of this technique, the minimum inhibitory concentrations (MICs) of 14 antimicrobial drugs against *E. coli* were evaluated (Table 2) using serial antibiotic dilutions. To achieve a turbidity corresponding to a McFarland standard of 0.5, overnight-grown bacteria were suspended in sterile phosphate-buffered saline (PBS, 7.4 pH). The solution was diluted by adding 100 µL of the suspension to 10 mL of Mueller-Hinton broth (Oxoid GmbH), yielding a concentration of roughly 10^6^–10^7^ colony forming units (cfu) per ml. A total of 100 µL of the suspension was pipetted in each well of the plate. The plates were then sealed and incubated aerobically for 18 to 24 h at 37 °C. At a wavelength of 620 nm, plates were read using a photometer (MICRONAUT, MERLIN Diagnostika GmbH, Bornheim, Germany). Growth was considered to be indicated by an optical density of >0.1. The German Institute for Standardization’s (DIN, Berlin, Germany) criteria were used to interpret MICs using the advanced expert system MCN-6 (Merlin). *E. coli* ATCC 25922, *E. coli* ATCC 35218, and *K. pneumoniae* ATCC 700603 were used as controls.

### 2.4. Identification of Colistin Resistance

The MICs for colistin resistance among isolates were determined using the RUO E-test colistin CO 256 according to the manufacturer’s instructions (bioMérieux Deutschland GmbH). To ensure uniform growth, Mueller-Hinton agar plates were equally streaked with an overnight bacterial suspension in Mueller-Hinton broth that had been adjusted to a density of McFarland 0.5. Once the agar surface dried, sterile forceps were used to apply an E-test^®^ colistin strip to the plate (concentration range: 0.016 to 256 g/mL). After 20 h of aerobic incubation at 37 °C, the MICs were identified as the location where the E-test strip was intersected by the suppression of bacterial growth. According to EUCAST’s clinical breakpoints, an isolate was deemed to be colistin-resistant if the MIC value was greater than 2 g/mL [17].

### 2.5. DNA Extraction and Purification

Genomic DNA was extracted from heat-inactivated pure cultures using the HighPure PCR Template Preparation Kit (Roche Diagnostics, Mannheim, Germany) according to manufacturer’s instructions. DNA quantity and purity were determined using a NanoDrop™ 1000 spectrophotometer (Thermo Fisher Scientific, Wilmington, NC, USA).

### 2.6. Confirmation of E. coli Isolates Using PCR

The confirmation of an isolate as *E. coli* was applied using a specific PCR assay targeting 16S rRNA-DNA genes as described in a previous study [29]. The PCR reaction was carried out with the following thermal profile: An initial denaturation step at 96 °C for 60 s was followed by 35 cycles of denaturation (96 °C for 15 s), annealing (58 °C for 60 s), and extension (72 °C at 45 s) with a final extension at 72 °C for 60 s. PCR amplicons of 585 bp were analyzed on a 1.5% non-denaturing agarose gel (PEQGOLD UNIVERSAL-AGAROSE, VWR, Mönchweiler, Germany), stained with ethidium bromide and visualized under UV light (Syngene G: BOX Chemi XT4, VWR, Germany). 

### 2.7. Microarray-Prediction of Genoserotypes of E. coli Isolates

The *E. coli* SeroGenoTyping AS-1 Kit (Abbott Technologies GmbH, Jena, Germany) and the DNA microarray-based assay CarbaResist from INTER-ARRAY (fzmb GmbH, Bad Langensalza, Germany), were used to determine the genotypes of *E. coli* isolates. Five microliters of extracted RNA-free high-quality DNA (with a concentration of at least 100 ng/μL) were labeled with biotin by a primer extension amplification using *E. coli* SeroGenoTyping AS-1 Kit according to manufacturer’s instructions. The procedures for multiplex labelling, hybridization, and data analysis were carried out as described in previous studies [30]. 

### 2.8. Detection of Carbapenemase Genes Using Multiplex Real-Time PCR

A multiplex real-time PCR detection of the most common carbapenemase genes *bla*KPC, *bla*NDM, *bla*VIM, and *bla*OXA-48 was carried out for all *E. coli* isolates. Primers and probes sequences are included in Table 2. PCR condition was initiated by initial denaturation at 95 °C for 4 min, followed by 50 cycles of denaturation at 95 °C for 30 s, annealing at 50 °C for 30 s and elongation at 72 °C for 60 s and final elongation step at 72 °C for 5 min. *Klebsiella* (*K.*) *pneumoniae, Citrobacter* (*C.*) *freundii, Pseudomonas* (*P.*) *aeruginosa,* and *E. coli* were positive controls for the reaction. CTs ≥ 40 were considered negative [31]. 

### 2.9. Statistical Analysis

A correlation analysis was performed to determine the association of antimicrobial resistance genes belonging to the same antimicrobial class or different classes among the examined isolates. Binary data (0/1) that denote absence/presence of resistance genes were imported into an R software (version 3.6.1; https://www.r-project.org, (accessed on 20 May 2022). The correlations were then calculated with package “corrplot” using the function “cor” and “cor.mtest” at a significance of *p* < 0.05.

## 3. Results

### 3.1. Isolation and Identification of E. coli

Out of 250 samples cultivated on Brilliance^TM^ ESBL agar, 26 suspicious isolates were confirmed as *E. coli* by MALDI-TOF and conventional PCR.

### 3.2. Antimicrobial Susceptibility Tsting

The results of the phenotypic antimicrobial susceptibility tests of *E. coli* isolates are illustrated in Table 3. *E. coli* isolates were all resistant to penicillin, rifampicin, streptomycin, and erythromycin. The resistance rates to tetracycline and trimethoprim/ sulfamethoxazole were 92.3%, each. All isolates were sensitive to imipenem. 

All eight isolates from young poults (6–10 days) were resistant to penicillin, streptomycin, tetracycline, erythromycin, chloramphenicol, rifampicin, and trimethoprim/ sulfamethoxazole (8/8, 100%). Additionally, 75% of them showed high MICs to ciprofloxacin, levofloxacin, and amoxicillin-clavulanic acid, respectively. The resistance rate to ceftazidime and gentamicin among isolates was 62.5%, each.

A total of 17 *E. coli* isolates (65.4%) were confirmed phenotypically as ESBL-producers. Among all isolates, only one *E. coli* isolate (3.8%) was phenotypically resistant to colistin.

### 3.3. Serogenotyping of E. coli Isolates Using Microarray Analysis

Serogenotyping by microarray revealed that seven out of 26 *E. coli* isolates (26.9%) belonged to O9, 27, 29, 112, 123, 126, and 141. Other isolates could not be assigned, most likely because several O-serotypes were not covered by the assay. 

H-antigen serotypes were identified in all isolates. Eleven different types of H-antigens were detected. H32 was the most common type, it was identified in four isolates, followed by H38 and H05 (two isolates each), while H37, 45, 31, 25, 30, 19, 04, and 10 were identified in only single isolates.

### 3.4. Detection of Antimicrobial Resistance and Virulence Determinants in E. coli by Microarray Analysis

Several resistance genes were identified in *E. coli* isolates using microarray-based analyses (Table 4). The 26 isolates originated from five provinces (Dakahliya (*n* = 12), Damietta (*n* = 8), Sharkiya (*n* = 3), Kafr El-Sheikh (*n* = 2), and Gharbiya (*n* = 1)). Microarray analysis revealed the presence of the beta-lactam resistance associated genes; *bla*TEM (*n* = 21; 80.8%), *bla*CTX-M9 (*n* = 6; 23,1%), in addition to *bla*SHV (*n* = 5; 19.2%) and *bla*OXA10 (*n =* 4; 15.4%).

With regard to other antimicrobial classes, the genes *str*A (*n* = 18; 69.2%), *aad*A1 (*n* = 17; 65.4%), *str*B (*n* = 17; 65.4%), *aad*A2 (*n* = 14; 53.8%) and *aph*A (*n* = 13; 50.0%) were the most frequently detected aminoglycoside resistance genes. Genes associated with sulfonamide resistance were *sul*1(*n* = 12; 46.2%), *sul*2 (*n* = 18; 69.2%), and *sul*3 (10; 38.5%). Macrolide resistance associated genes were *mph* (*n* = 14; 53.8%) and *mrx* (*n* = 9; 34.6%).

Chloramphenicol resistance associated genes were *flo*R (*n* = 17; 65.4%) and *cml*A1 (*n* = 12; 46.2%). Tetracycline and quinolone-associated resistance gene *tet*A and *qnr*S were detected in approximately half of *E. coli* isolates (*n* = 15; 57.7%) and (*n* = 13; 50.0%). 

Regarding genes encoding virulence factors, the class 1 Integron integrase *int*I1 gene was detected in 22 (84.6%), while transposase gene for the transposon *tnp*ISEcp1 was detected in 9 (34.6%) and OqxB—integral membrane protein, component of RND-type multidrug efflux pump *oqx*B in 2 (7.7%) isolates.

Additionally, the colistin resistance associated gene *mcr*-9 was identified in one isolate (3.8%).

All *E. coli* isolates obtained from young poults (6–10 days) harbored *bla*TEM. Additionally, one or more of the following resistance genes were identified in these isolates; *bla*SHV, *bla*CTX-M9 and *bla*OXA-10. The class 1 integron integrase *int*I1 (84.6%), transposase for the transposon *tnp*ISEcp1 (34.6%) and OqxB -integral membrane and component of RND-type multidrug efflux pump *oqx*B (7.7%) were identified among the isolates. Genes encoding virulence factors including fimbrae (*ipf*A), toxins (*ast*A and *cma*), in addition to miscellaneous virulence-associated genes (*int*l1, *hem*L, *iro*N, *iss*) were identified among isolates.

Most of resistance and virulence genes against other antimicrobial classes were also identified among these isolates (Table 4 and Table S1).

### 3.5. Association of Antimicrobial Resistance Genotypes among the Examined Isolates

**Figure 1:**
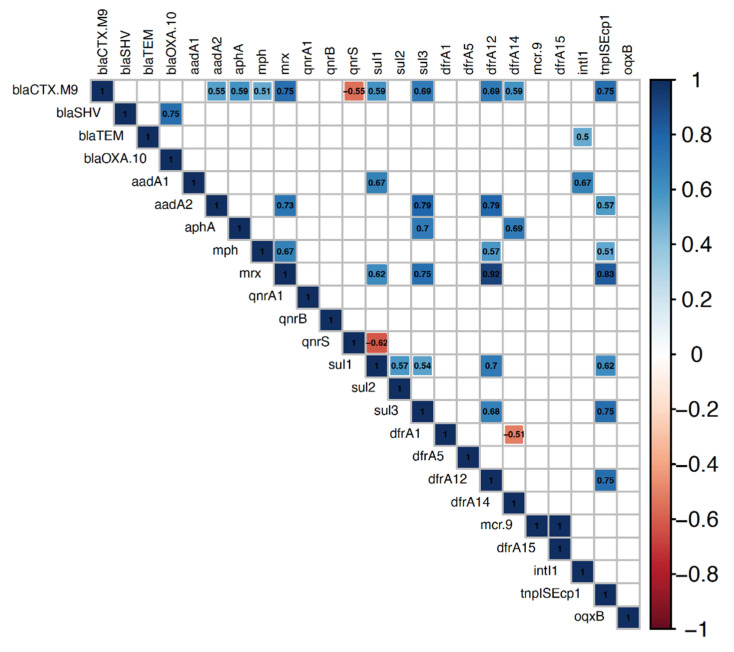
Correlation analysis determines the associations between resistance genes among *E. coli* isolates from turkeys. The blue and red boxes indicate positive and negative correlations, respectively. The strength of color corresponds to the numerical value of the correlation coefficient (*r*). Significance was calculated at *p* < 0.05, and boxes with non-significant correlations were left blank.

The associations between resistance genes either those belonged to the same antimicrobial class or different classes were determined among the examined isolates using correlation analysis. Positive correlations were observed for pairs of resistance genes belonging to the same class, i.e., *bla*SHV and *bla*OXA-10 (r = 0.75), *sul*1 with *sul*2 (r = 0.57) and *sul*3 (r = 0.54), *mph* and *mrx* (r = 0.67). Positive correlations were also found for the co-occurrence of resistance genes from different antibiotic classes. For instance, *bla*CTX-M-9 showed positive significant associations with aminoglycosides (*aad*A2, *aph*A), macrolide (*mph*, *mrx*), sulfonamides (*sul*1, *sul*3), and dihydrofolate reductase (*dfr*A12, *dfr*A14) resistance genes. On the other hand, negative correlations were observed for genes belonging to the same antimicrobial class (*dfr*A1 and *dfr*A14) as well as genes belonging to different antimicrobial classes (*bla*CTX-M-9 and *qnr*S; *sul*1 and *qnr*S) Figure 1.

## 4. Discussion

Commensal *E. coli* is a normal inhabitant that maintains the normal gut microbiota in poultry. However, avian pathogenic strains (APEC) mostly carry virulence genes and cause extraintestinal infections in birds. Colibacillosis is mostly caused by APEC, including systemic and localized infections such as omphalitis, swollen head syndrome, cellulitis, diarrhea, respiratory colisepticaemia and enteric colisepticemia [17,32]. Furthermore, it could have a zoonotic pathogenic potential such as neonatal meningitis [33]. The infection of *E. coli* in poultry farms can spread both vertically and horizontally. It can spread directly by contaminated dust, water, fomites, and feces. *E. coli* can be inhaled or consumed to cause infection, resulting in illness. High ammonia concentrations, which can harm the skin or respiratory epithelium and make it easier for *E. coli* to enter the body, are among the most significant non-infectious predisposing variables. The prevalence and severity of *E. coli* infections are consequently increased by these factors in addition to poor hygiene, short distances between houses, flocks of different ages on a farm, and short service intervals between flocks. *E. coli* can cause omphalitis and inflammation of the yolk sac when it is vertically transmitted from parent flocks to their offspring. Additionally, it can be transmitted between chicks during hatching and is typically linked to a high mortality rate whenever the yolk sacs become inflamed. Instead of actual vertical transmission within the egg, contamination of the eggshell is mostly to blame for this sequence. Hygiene and management practices are part of the control regimens to stop both the introduction and spread of infection. Longer downtimes between flocks, improved air quality, and thorough cleaning and disinfection are other additional approaches to be considered [34].

Previous studies reported high prevalence of antibiotic resistance rates among APEC in turkeys [15,33,35].

In this study, 26 *E. coli* were isolated from 250 cloacal swabs collected from 12 turkey farms located in 5 governorates in northern Egypt. The samples were investigated for the presence of ESBL and/or carbapenemase producing *E. coli.* The *E. coli* isolates were confirmed either as ESBLs or non ESBLs producers through phenotypic resistance to Ceftazidime. This was a high percentage in healthy turkeys in comparison with a previous result of only 2.2% ESBL producing *E. coli* isolates from turkeys in other studies [4].

Antimicrobials are usually used in turkey hatcheries to prevent omphalitis in newly hatched birds [36]. Few antimicrobials are available for this purpose, mainly third generation cephalosporins and most are used off-label [37].

In the current investigation, all isolates were MDR, defined as resistant to three or more antimicrobial classes, which is alarming.

Ceftazidime, a third-generation cephalosporin has a high importance in human medicine and it is used to treat severe infections. It is a major concern that the use of ceftazidime in food animals can promote resistance to other cephalosporins, such as ceftriaxone, used in humans and vice versa [38]. Although cephalosporins are not approved for use in poultry, the administration of these drugs in poultry and turkey farms is an emerging problem. Resistance to third and fourth-generation cephalosporins in fecal turkey *E. coli* has been previously reported [15,35,36,39,40,41,42]. In this study, the prevalence of ceftazidime resistance among all isolates was 65.4%, while its prevalence in young poults (6–10 days) was 62.5%, which is certainly high.

Gentamicin, an aminoglycoside, is classified as a drug of high importance to humans. In this study, the resistance rate to the drug was found in 65.4% of all isolates and in 62.5% of isolates derived from young poults. 

It was reported that the highest rates of resistance among pathogenic and commensal *E. coli* in turkeys were detected against tetracyclines, penicillins, and sulfonamides [15,40]. The high levels of resistance to these classes may be attributed to a long-term selection pressure, as these antimicrobials are very old and widely used in the poultry industry all over the world [43]. The resistance to tetracycline amongst *E. coli* is suggested to be selected by a bystander effect on commensal *E. coli*, during treatment of other Enterobacteriales. Bacterial resistance to tetracycline is most commonly mediated by energy-dependent pumping of tetracycline out of the bacterial cell. The *tet*(A), -(B), -(C), -(D), -(E), -(Y), and -(I) genes in Gram-negative bacteria encode such efflux systems [44].

In this study, the isolates showed resistance prevalence of 88.5% to chloramphenicol, 92.3% for both tetracycline and trimethoprim/sulfamethoxazole and a full phenotypic resistance prevalence (100%) to penicillin, streptomycin, erythromycin and Rifampicin, respectively. Additionally, all isolates from young poults were fully resistant to penicillin, streptomycin, tetracycline, erythromycin, chloramphenicol, rifampicin, and trimethoprim/ sulfamethoxazole. This result was in accordance with a previous study which reported a high resistance to tetracycline, streptomycin, and sulfisoxazole in *E. coli* isolated from turkey hatcheries [37]. Moreover, *E. coli* isolated from turkeys previously were highly resistant to tetracyclines, penicillins, and sulfonamides [45,46]. 

Erythromycin resistance is intrinsic in *E. coli* and the members of the erythromycin esterase family known as *ere*A and *ere*B, were first discovered in clinical *E. coli* strains [47].

It has been recognized that fluoroquinolone resistance can be related to the usage of these drugs in a given country, which is in turn influenced by the market dynamics, mainly the cost of commercial products [48]. In this study, most ESBL producing *E. coli* isolates showed additional high resistance prevalence to fluoroquinolones, aminoglycosides, chloramphenicol, tetracycline, macrolides, and ansamycins. In a previous study, an additional high resistance prevalence to aminoglycosides, sulfonamides, and tetracyclines was recorded among ESBL producing *E. coli* isolates in turkeys [46].

The resistance to the majority of antimicrobials tested in this study can be attributed to the acquirement of foreign resistance genes through horizontal transfer, which usually involves both pathogenic and commensal bacteria [49,50]. 

In this study, resistance genes associated with beta-lactam resistance were *bla*TEM (80.8%), *bla*CTX-M9 (23.1%), *bla*SHV (19.2%) and *bla*OXA-10 (15.4%). All *E. coli* isolates of young poults (6–10 days), harbored *bla*TEM, while other beta-lactam resistance genes, CTX-M9, *bla*SHV and OXA-10 were found in 37.5%, 12.5 and 12.5% *E. coli* isolated from young poults, respectively.

These results were higher than other previous studies, which identified *bla*CTX-M8, *bla*CTX-M-2 and *bla*TEM in 6.8, 31, and 70% of ESBL-producing *E. coli* isolated from turkey flocks showing signs of colibacillosis [51]. The prevalence of *bla*CTX-M2 was only 1% in *E. coli* isolated from turkeys with air-sacculitis in previously reported results [52], and also (*bla*CTX-M-1, *bla*CTX-M-2, and *bla*SHV-12) were detected in 7, 9, and 4% of isolates from turkeys [46].

The colistin resistance associated *mcr-*1 gene was detected first in 2015 in raw meat, livestock and human-originated samples in China. The latest *mcr* gene variant, *mcr*-9, was reported first in 2019. This allele shares about 64.5% amino acid identity with *mcr*-3 [53]. It was previously reported that phenotypic colistin resistance in *E. coli* is mostly associated with the carriage of *mcr-*1 gene [54].

In this study, one *E. coli* isolate (3.8%) was phenotypically colistin-resistant and harbored *mcr*-9 gene. The *mcr-*1 gene was previously reported in *E. coli* isolates from healthy broiler chickens in Egypt [17].

The *mcr*-1 gene was identified in randomly collected *E. coli* isolates from pigs, poultry, and turkeys in France with 0.5, 1.8, and 5.9%, respectively [55], and 5.05% of phenotypically colistin-resistant *E. coli* isolates from broilers in Germany [56].

Bacterial efflux pumps (EPs) are proteins that are positioned and embedded in the bacterium’s plasma membrane. Their purpose is to recognize toxic substances that have gotten past the organism’s protective cell wall and into the periplasm or cytoplasm and extrude them before they reach their target organelles. They are mostly described as a major mechanism of drug resistance [57]. A common form of drug-resistance emergence in Enterobacteriales is mediated by transposons. *E. coli* is known to harbor a wide range of transposons such as Tn3, Tn5, Tn7, Tn9, and Tn10, that are all related to antimicrobial resistance ampicillin, kanamycin, trimethoprim, spectinomycin, streptomycin, chloramphenicol, and tetracycline [58].

The transmission of resistance genes in bacteria has been linked to a number of acquired resistance mechanisms in recent years, including bacteriophages, transposons, plasmids, and integrons. Integrons are one of the genetic components that may have a role in the widespread occurrence and dissemination of antibiotic resistance [59]. These components have the ability to seize, incorporate, and mobilize antibiotic resistance gene cassettes. Based on the genetic similarity of the integrase *int*I gene sequence, the integrons were divided into three major groups, namely 1, 2, and 3. Class 1 integrons are more prevalent in Gram-Negative bacteria. Metallo-beta-lactamase (MBL) and class 1 integron are mostly encoded on the same gene cassettes that are circulating in bacterial populations [60]. Furthermore, newly extended spectrum beta lactamases (ESBL)-encoding genes such as *bla*CTX-M, *bla*GES, and *bla*VEB-1 are typically found on integron-like structures [61].

In the current study, the class 1 Integron integrase *intI*1 gene was detected in 84.6% among *E. coli* isolates. Moreover, transposase gene for the transposon *tnp*ISEcp1 and OqxB - integral membrane protein, component of RND-type multidrug efflux pump *oqx*B were identified in 34.6% and 7.7% of isolates.

Similarities between human and avian *E. coli* virulence-associated factors were previously reported in many studies [62]. Although the *E. coli* isolates in this study were commensal, they harbored genes encoding virulence factors including fimbrae (*ipf*A), toxins (*ast*A and *cma*) that are shared between avian and human-isolated *E. coli*, in addition to miscellaneous virulence-associated genes (*intl*1, *hem*L, *iro*N, *iss*). These similarities could emphasize a human transmission origin.

## 5. Conclusions

The obtained results strengthen the need to develop surveillance strategies and control procedures to reduce the use of antibiotics and subsequently the development of antimicrobial resistance.

Understanding how an ESBL and/or carbapenemase gene mobilizes through a bacterial population will be critical for detection methods and ultimately inform infection control practices. Additional understanding of gene mobilization and tracking will require novel approaches to surveillance, which will be required to slow the spread of this emerging resistance.

The strategies to contain AMR emergence rely on a robust antibiotic surveillance system, assessing the threats imposed due to the emergence of MDR.

## Figures and Tables

**Table 1:** Investigated turkey farms in northern Egypt and number of collected samples.

Numbers	Governorates					Total
	Dakahliya	Damietta	Kafr El-Sheikh	Sharkiya	Gharbiya	5
Farms	4	3	2	2	1	12
Bird capacity	5000	2100	1200	1800	800	10,900
Samples	71	44	46	46	43	250

**Table 2:** Target genes, primers and probes.

Target Gene	Primer, Probe	Nucleotide Sequence (5′-3′)	Fragment Length (bp)
*bla*_KPC_	KPC-FW	CTG TAT CGC CGT CTA GTT CTG	101
	KPC-RV	AGT TTA GCG AAT GGT TCC G	
	KPC-P	6FAM- TGT CTT GTC TCT CAT GGC CGC TGG –BHQ1	
*bla*_NDM-1_	NDM-FW	GCA TTA GCC GCT GCA TT	100
	NDM-RV	GAT CGC CAA ACC GTT GG	
	NDM-P	ROX- ACG ATT GGC CAG CAA ATG GAA ACT GG –BHQ2	
*bla*_VIM_	VIM-FW	TGG CAA CGT ACG CAT CAC C	70
	VIM-RV	CGC AGC ACC GGG ATA GAA	
	VIM-P	Cy5- TCT CTA GAA GGA CTC TCA TCG AGC GGG–BHQ3	
*bla*_OXA-48_	OXA-48-FW	TTC CCA ATA GCT TGA TCG C	143
	OXA-48-RV	CCA TCC CAC TTA AAG ACT TGG	
	OXA-48-P	HEX- TCG ATT TGG GCG TGG TTA AGG ATG AAC–BHQ1	

**Table 3:** Phenotypic resistance profiles of *E. coli* isolates detected by broth microdilution.

Antibiotic	0.03125	0.0625	0.125	0.25	0.5	1	2	4	8	16	32	64	128	256	Resistant	%
Trimethoprim/Sulfamethoxazole (T/S)	1/19 (2)	2/38	4/76 (24)												24	92.3
Penicillin (PEN)					26										26	100
Streptomycin (STR)									4	22					26	100
Amoxicillin/Clavulanic acid (AMC)					0.5/0.25	1/0.5 (1)	2/1	4/2 (3)	8/4 (3)	16/8 (10)	32/16 (5)	64/32 (3)			18	69.2
Ceftazidime (CAZ)					2		2	4	4	3	10				17	65.4
Imipenem (IMP)			18	1	5										0	0
Ciprofloxacin (CIP)				7	1		6	12							18	69.2
Levofloxacin (LEV)				4	4		1	17							18	69.2
Gentamicin (GEN)					2	1	3	1	1	2	14				17	65.4
Amikacin (AMK)						1	8	12	3			2			2	7.7
Tetracycline (TET)						1			1	24					24	92.3
Erythromycin (ERY)									1	25					26	100
Chloramphenicol (CMP)							1	1	1		2	21			23	88.5
Rifampicin (RAM)								26							26	100

Concentration ranges provided for each antimicrobial drug, are presented in shadows.

**Table 4:** Phenotypic and genotypic resistance profiles of *E. coli* isolates from healthy turkeys in five provinces in Egypt.

District	Isolate Code	Age (d)	Virulence Genes	Resistance Genes	Phenotypic Resistance
Dakahliya	CS0284-1	365	*hem*L, *tnp*ISEcp1	*strA, strB, cmlA1, floR*	PEN, STR, AMC, ERY, RAM
	CS0302-3	365	*intl1*	*bla*TEM, *aadA2, aphA, qnrS, sul3, tetA, strA, strB, cmlA1, floR, dfr*A1, *dfr*A15, *mcr*-9	PEN, STR, CAZ, CIP, LEV, GEN, AMK, TET, ERY, CMP, RAM, T/S
	CS0290-2	365	*intl1*	*tetA, strA, strB, cmlA1, floR, aadA1, sul1, sul2, dfrA1*	PEN, STR, AMC, CAZ, CIP, LEV, GEN, TET, ERY, CMP, RAM, T/S
	CS0299-1	6	*intl1*	*bla*TEM*, aphA, mph, qnrS, dfrA14, tetA, strA, strB, cmlA1, floR,*	PEN, STR, CAZ, CIP, LEV, GEN, AMK, TET, ERY, CMP, RAM, T/S
	CS0303-1	6	*hem*L, *intl*1	*bla*SHV*, bla*TEM, *bla*OXA-10, *aadA1, aadA2, mph, qnrS, sul2, dfrA14, dfrA12, dfrA14, tetA, strA, strB, cmlA1, floR*	PEN, STR, AMC, CAZ, CIP, LEV, GEN, TET, ERY, CMP, RAM, T/S
	CS0298-2	6	*ast*A, *hem*L, *intl*1, *tnp*ISEcp1	*bla*CTX-M9, *bla*TEM, *aadA1, aadA2, aphA, mph, mrx, sul1, sul2, sul3,tet*A, *str*A, *str*B, *cmlA1, floR*	PEN, STR, AMC, CAZ, CIP, LEV, GEN, TET, ERY, CMP, RAM, T/S
	CS0290-1	365	*hem*L	*bla*SHV, *bla*TEM, *qnrS, strA, strB, floR, arr*	PEN, STR, CAZ, GEN, ERY, CMP, RAM
	CS0296-2	6	*hem*L, *intl*1, *tnp*ISEcp1	*bla*CTX-M9, *bla*TEM, *aad*A1, *aad*A2, *aph*A, *mrx*, *sul1, sul2, sul3, dfrA12, dfr*A14, *tetA, strA, strB, cmlA1, floR*	PEN, STR, AMC, CAZ, CIP, LEV, GEN, TET, ERY, CMP, RAM, T/S
	CS0278-2	365	*intl1, oqxB*	*bla*TEM, *aphA, qnrS, sul2, dfrA14, tetA, aar, cmlA1*	PEN, STR, CAZ, TET, ERY, CMP, RAM, T/S
	CS0304	6	*ifp*A, *cma*, *hem*L, *intl*1	*bla*TEM, *aadA1, aadA2, aphA, qnrS, sul3, dfrA14*	PEN, STR, AMC, TET, ERY, CMP, RAM, T/S
	CS0305-1	6	*ast*A, *hem*L, *intl*1, *tnp*ISEcp1	*blaC*TX-M9, *bla*TEM, *aadA1, aadA2, aphA, strA, strB, mrx, cmlA1, floR, sul1, sul2, sul3, dfrA12, dfrA14*	PEN, STR, TET, ERY, CMP, RAM, T/S
	CS0310-1	365	*ifp*A, *hem*L, *iss, intI1*	*mph, floR, arr*	PEN, STR, TET, ERY, RAM, T/S
Damietta	CS0294-1	240		*aadA1, strA, strB, mph, sul1, sul2, dfrA1, arr, higA, tetA,* *cmlA1, floR*	PEN, STR, CAZ, CIP, LEV, GEN, TET, ERY, CMP, RAM, T/S
	CS0317	240	*cma*, *intl*1, *hem*L, *iro*N, *iss*	*bla*TEM*, aadA1, strB, mph, qnrS, sul2, tetA, dfrA1, floR, cmIA1*	PEN, STR, AMC, CAZ, CIP, LEV, TET, ERY, RAM, T/S
	CS0314-1	240	*intI1, tnpI*SEcp1	*bla*CTX-M9*, bla*TEM*, aadA1, aadA2, aphA, strA, strB, mph, mrx, sul1, sul2, sul3, dfrA12, dfrA14,*	PEN, STR, AMC, CAZ, CIP, LEV, GEN, TET, ERY, CMP, RAM, T/S
	CS0316-1	240	*intl1, oqxB*	*bla*TEM, *aadA1, mph, qnrS, dfrA1*	PEN, STR, AMC, CAZ, CIP, LEV, TET, ERY, CMP, RAM, T/S
	CS0324-2	240	*intl*1, *hem*L	*bla*TEM, *aadA2, strA, strB, floR, mph, mrx, qnrS, tetA, sul2, dfrA12, dfrA5*	PEN, STR, CIP, LEV, GEN, TET, ERY, CMP, RAM, T/S
	CS0328-3	240	*hem*L	*tet* *A, mph*	PEN, STR, AMC, CIP, LEV, GEN, TET, ERY, CMP, RAM, T/S
	CS0329	240	*ast*A, *intl*1, *hem*L*, tnp*ISEcp1	*bla*CTX-M9, *bla*TEM*, aadA1, aadA2, aphA, strA, strB, mph, mrx, sul1, sul2, sul3, cmlA1, floR, tetA, dfrA14, dfrA12*	PEN, STR, AMC, CAZ, CIP, LEV, GEN, TET, ERY, CMP, RAM, T/S
	CS0332-2	10	*intl*1, *hem*L	*bla*TEM, *aphA,strA, strB, qnrS, sul2, dfrA14, cmIA1, floR*	PEN, STR, AMC, CIP, LEV, TET, ERY, CMP, RAM, T/S
Gharbiya	CS0281-2	365	*int*I1	*bla*TEM, *bla*OXA-10, *aadA1, strA, strB, qnrA1, sul1, sul2, floR*	PEN, STR, GEN, TET, ERY, CMP, RAM, T/S
Kafr El-sheikh	CS0296-1	365	*int*I1	*bla*SHV, *bla*TEM, *bla*OXA10, *aadA1, aadA2, strA, cmlA1, floR, qnrS, arr, tetA, sul2, dfrA14*	PEN, STR, AMC, TET, ERY, CMP, RAM, T/S
	CS0408-2	150	*intl*1, *ifp*A, *hem*L, *iss,tnp*ISEcp11	*bla*SHV, *bla*TEM, *aadA1, aadA2, mph, mrx, aphA, qnrS, sul1, sul2, sul3, dfrA12, dfrA14*	PEN, STR, AMC, CAZ, GEN, TET, ERY, CMP, RAM, T/S
Sharkiya	CS0319-1	240	*intI*1*, tnp*ISEcp1	*bla*TEM*, bla*CTX-M9, *aadA1, aadA2, aphA, strA, strB, mph, mrx, sul1, sul2, sul3, dfrA12, dfrA14, tetA*	PEN, STR, AMC, CAZ, CIP, LEV, GEN, TET, ERY, CMP, RAM, T/S
	CS0336-2	10	*int*I1	*bla*TEM, *aadA1, aadA2, strA, qnrB, sul1, sul2, dfrA1, dfrA12, tetA*	PEN, STR, AMC, CAZ, CIP, LEV, GEN, TET, ERY, CMP, RAM, T/S
	CS0357-2	75	*intI1, tnp*ISEcp1	*bla*SHV*, bla*TEM, *bla*OXA10*, aadA1, aadA2, aphA, mph, mrx, qnrS, sul1, sul2, sul3, dfrA12*	PEN, STR, AMC, CAZ, CIP, LEV, TET, ERY, CMP, RAM, T/S

## Supplementary Materials

The following are available online at https://www.mdpi.com/article/10.3390/antibiotics11081075/s1, Table S1: Phenotypic and serogenotypic characteristics of *E. coli* isolates.
